# The possible role of SRMS in colorectal cancer by bioinformatics analysis

**DOI:** 10.1186/s12957-021-02431-y

**Published:** 2021-11-16

**Authors:** Jie Zhang, Weidong Liu, Sisi Feng, Baiyun Zhong

**Affiliations:** 1 Department of Clinical Laboratory, Xiangya Hospital, Central South University, 87 Xiangya Road, Kaifu District, Changsha, 410008 China; 2 Department of Essential Surgery, Xiangya Hospital, Central South University, Changsha, China

**Keywords:** Srms, Colorectal cancer, Immune signatures, Bioinformatics analysis

## Abstract

**Background:**

Src-related kinase lacking C-terminal regulatory tyrosine and N-terminal myristoylation sites (SRMS) is a non-receptor tyrosine kinase that has been found to be overexpressed in various tumors. However, the role of SRMS in colorectal cancer (CRC) has not been well established.

**Methods:**

We evaluated the expression levels of SRMS in CRC using GEPIA, Oncomine, and HPA datasets. Survival information and gene expression data of CRC were obtained from The Cancer Genome Atlas (TCGA). Then, the association between SRMS and clinicopathological features was analyzed using UALCAN dataset. LinkedOmics was used to determine co-expression and functional networks associated with SRMS. Besides, we used TISIDB to assess the correlation between SRMS and immune signatures, including tumor-infiltrating immune cells and immunomodulators. Lastly, protein-protein interaction network (PPI) was established and the function enrichment analysis of the SRMS-associated immunomodulators and immune cell marker genes were performed using the STRING portal.

**Results:**

Compared to normal colorectal tissues, SRMS was found to be overexpressed in CRC tissues, which was correlated with a poor prognosis. In colon adenocarcinoma (COAD), the expression levels of SRMS are significantly correlated with pathological stages and nodal metastasis status. Functional network analysis suggested that SRMS regulates intermediate filament-based processes, protein autophosphorylation, translational initiation, and elongation signaling through pathways involving ribosomes, proteasomes, oxidative phosphorylation, and DNA replication. In addition, SRMS expression was correlated with infiltrating levels of CD4+ T cells, CD56dim, MEM B, Neutrophils, Th2, Th17, and Act DC. The gene ontology (GO) analysis of SRMS-associated immunomodulators and immune cell marker genes showed that they were mainly enriched in the immune microenvironment molecule-related signals. Kyoto Encyclopedia of Genes and Genomes (KEGG) enrichment analysis of these genes indicated that they are involved in multiple cancer-related pathways.

**Conclusions:**

SRMS is a promising prognostic biomarker and potential therapeutic target for CRC patients. In particular, SRMS regulates CRC progression by modulating cytokine-cytokine receptor interaction, chemokines, IL-17, and intestinal immune networks for IgA production signaling pathways among others. However, more studies are needed to validate these findings.

## Introduction

Globally, CRC is one of the most prevalent malignancies and the fourth leading cause of cancer-related mortality, resulting in almost 900,000 annual mortality [1]. The high incidence of colorectal cancer is correlated with age, diet, race, lifestyle, genetic alteration, and other factors. Since the early symptoms of colorectal cancer are not typical, about 25% of colorectal cancers present distant metastasis at the time of initial diagnosis [2]. Currently, the major therapeutic options for CRC include surgery, chemotherapy, radiotherapy, biotherapy, and immunotherapy. However, the 5-year survival outcomes for advanced CRC patients are approximately 10% [3, 4]. Identification of genes associated with tumor formation and metastasis will provide new ideas and targets for anti-CRC therapy.

Src-related kinase lacking C-terminal regulatory tyrosine and N-terminal myristoylation sites (SRMS) encode a 53-kDa non-receptor tyrosine kinase protein that was first cloned in mouse neural precursor cells [5]. SRMS is composed *of* 488 amino acids, which *ha*s a similar structure to Src family kinases. Its functional regions are composed of a Src homology 3 (SH3) domain, a Src homology 2 (SH2) domain, and a protein kinase domain [6]. These domains are important in mediating a series of intra-molecular or inter-molecular interactions as well as downstream signal transductions. The non-receptor tyrosine kinase plays an important role in the regulation of cell growth, proliferation, and invasion by activating the downstream substrate to initiate tyrosine phosphorylation [7]*.* Compared with other members of the non-receptor tyrosine kinase family, the roles and functions of SRMS are still in the early stages. A recent review article noted that SRMS was overexpressed in six breast cancer cell lines and its levels were elevated in breast tumors compared to adjacent normal tissues [8]. In addition, a proteomic study of gastric cancer patients showed that SRMS was the only differentially expressed kinase [9]. However, the expression levels and biological functions of SRMS in CRC have not been clearly elucidated.

In this study, we evaluated and validated the expression levels and prognosis value of SRMS in CRC through multiple independent cohorts. Then, the relationship between SRMS expression levels and clinic-pathological features such as histological grade and metastasis were systemically determined. In addition, we performed co-expression analysis and assessed the gene sets associated with SRMS in CRC through gene set enrichment analysis (GSEA). Lastly, we assessed the roles of SRMS in tumor immunity.

## Materials and methods

### *SRMS differential expression and* proggesnosis *analysis*

GEPIA (http://gepia.cancer-pku.cn/) is a database used to perform comprehensive and customizable functions using TCGA and GTEx data [10]. It includes 9736 tumor and 8587 normal samples. We used GEPIA to analyze the mRNA expression levels of SRMS in tumor and normal CRC tissue samples. The *Y*-axis represents the average log2 abundance in transcripts-per-million (TPM). DNA copy number variations (CNV) of SRMS in CRC and normal tissues were examined using the Oncomine 4.5 database. Oncomine (https://www.oncomine.org/) is the largest cancer microarray database and data-mining platform [11]. It contains 715 datasets and 86,733 samples. The screening conditions were set as ① gene: “SRMS”;② analysis type: “cancer VS normal analysis”; ③ cancer type: “Colorectal cancer”; ④ data type: “mRNA”; and ⑤ *P* value < 0.05, fold change > 2, gene rank = top 10%.

Subsequently, we compared SRMS protein expression levels between CRC tissues and normal colorectal tissues in the Human Protein Atlas (HPA) database. The HPA database (https://www.proteinatlas.org/) is aimed at mapping IHC-based protein expression profiles in cancerous and normal tissues as well as in cell lines [12]. Protein expression score is based on immunohistochemical data manually scored with regard to staining intensity (negative, weak, moderate, or strong) and fraction of stained cells (< 25%, 25–75%, or > 75%). For prognostic analysis, the gene expression profiles and clinical information for CRC patients were obtained from the TCGA database. Kaplan-Meier survival curves were plotted with R 3.6.3.

### UALCAN analysis

UALCAN (http://ualcan.path.uab.edu/) is a comprehensive and interactive web-portal that provides an easy access to publicly available cancer OMICS data [13]. Moreover, it has gene expression profiles and patient survival information. In this study, SRMS expression levels in various sub-groups of clinical characteristics (age, gender, race, stages, histological types, and nodal metastasis status) were examined in UALCAN. *p* ≤ 0.05 was considered statistically significant.

### LinkedOmics analysis

LinkedOmics (http://www.linkedomics.org/login.php) is a web-based platform that includes multiple omics data and proteomics data based on mass spectrometry (MS) generated by the CPTAC [14]. The “LinkFinder” module of LinkedOmics was used to evaluate gene co-expressions that are related to SRMS in the TCGA CRC cohort (*n* = 379). Statistical analyses were conducted using Pearson’s correlation coefficients and results were presented in form of heatmaps, scatter plots, and volcano plots. The expression of co-expressed genes was normalized using *Z*-score approach, *Z* = (*X*-mean (*X*))/sd (*X*). Based on the median SRMS gene expression, the data were divided into two groups, high expression group and low expression group. The color of the bar indicates the redder the color, the higher the gene expression; the bluer the color, the lower the gene expression.

In the “LinkInterpreter” module of LinkedOmics, we also performed functional enrichment analysis of co-expressed genes. Data from LinkFinder were used to perform GO and KEGG pathway enrichment analysis through GSEA. Rank criterion was an FDR < 0.05 and 500 simulations.

### TISIDB analysis

TISIDB (http://cis.hku.hk/TISIDB/) is a web portal for tumor and immune system interactions, including genomics and transcriptomics of 30 cancer types from TCGA, RNA sequencing data set of patient cohorts treated with immunotherapy [15]. The TCGA database provides a large amount of tumor public data, providing useful information for studying the complex interaction of the tumor microenvironment [16]. TISIDB has “Function,” “Literature,” “Screening,” “Immunotherapy,” “Lymphocyte,” “Immunomodulator,” “Chemokine,” “Subtype,” “Clinical,” and “Drug” modules. We used the TISIDB database to determine the correlations between SRMS expression levels and tumor-infiltrating lymphocytes (TILs) and immunomodulators.

### STRING analysis

STRING (https://string-db.org/) is a database of known and predicted protein-protein interactions [17]. Currently, it has 24,584,628 proteins from 5090 organisms. In this study, STRING was used to visualize protein-protein interaction networks and to predict the functions of the SRMS-associated immunomodulators and immune cell marker genes. GO and KEGG enrichment analysis was performed using Cluster-Profiler package 3.14.0. Proteins with a minimum required interaction score greater than or equal to 0.400.

## Result

### SRMS expression and prognosis in colorectal cancer

**Fig. 1:**
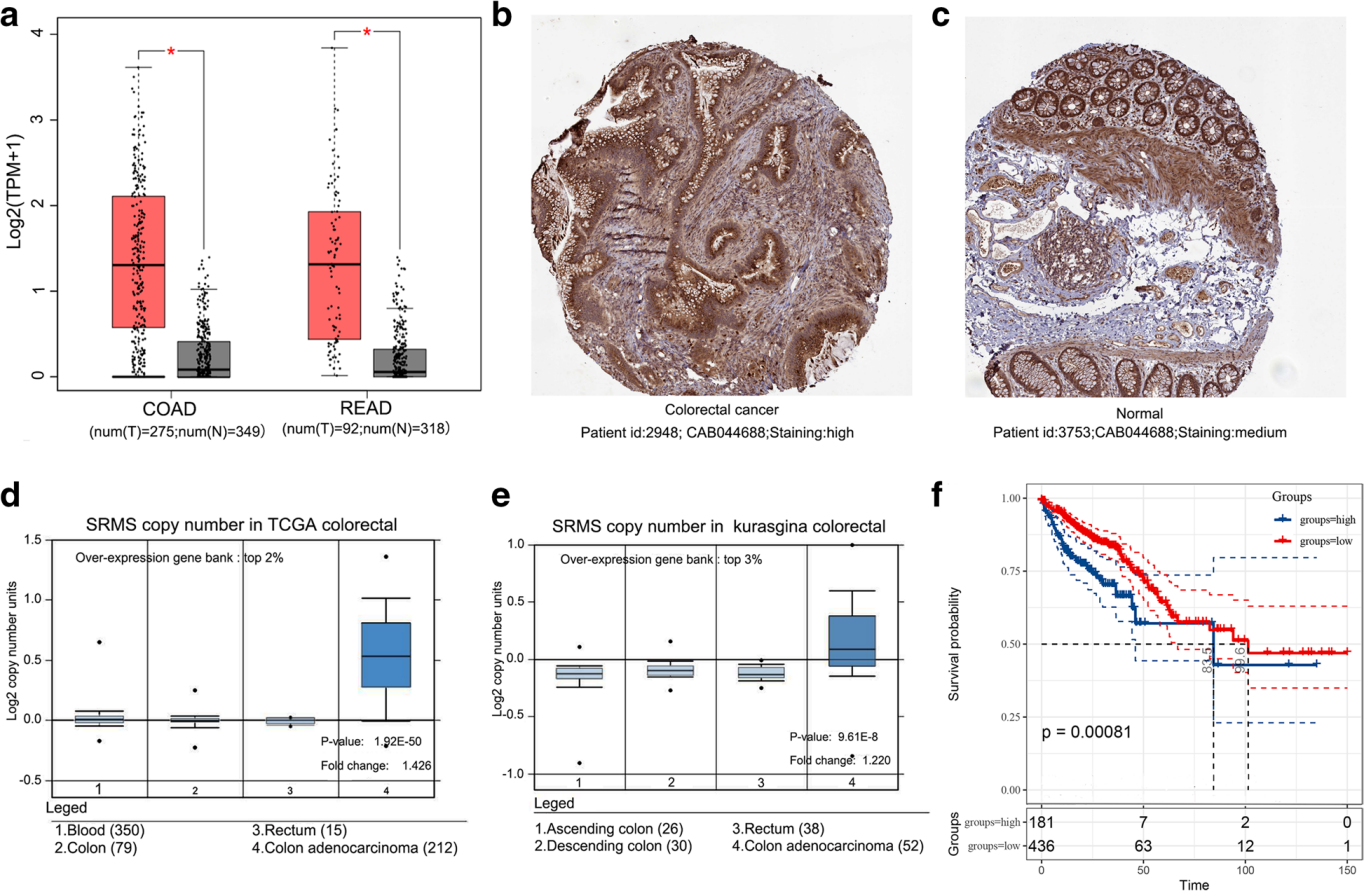
Expression and prognostic value of SRMS in CRC. **A** Comparison of SRMS mRNA expression levels between normal and tumor tissues in the TCGA and GTEx CRC cohorts, *p* value cutoff < 0.01, |Log2FC| Cutoff< 1. **B** Protein expression levels of SRMS in CRC tissues from the HPA database. **C** Protein expression levels of SRMS in non-cancerous colorectal tissues. **D** SRMS copy numbers in the Cancer Genome Atlas (TCGA) Colorectal datasets. *, *p* < 0.05. (E) SRMS copy numbers in Kurashina colorectal datasets. *, *p* < 0.05. **F** Kaplan-Meier survival curves reveal the prognostic value of SRMS in CRC

Initially, we compared mRNA expression levels of SRMS in GEPIA, which matched TCGA and GTEx data. mRNA expression levels of SRMS were significantly elevated in CRC tissues than in the paired adjacent normal tissues (*p* ≤ 0.05, Fig. 1A). Moreover, from the HPA database, protein expression levels of SRMS were high elevation in tumor tissues and moderate in normal tissues (Fig. 1B, C). Data in the Oncomine database, including TCGA colorectal and Kurasgina colorectal, showed that CNVs of SRMS were significantly elevated in colon adenocarcinoma (COAD) tissues than in paired normal tissues (*p* < 0.01, Fig. 1D, E). KM-Plotter shows that SRMS overexpressed is associated with a poor prognosis (Fig. 1F). It is worth noting that a more clear difference can be found between the overall survivals rates within 2000 days among the two groups. Therefore, SRMS expression is a potential diagnostic and prognostic indicator for CRC.

### SRMS expression in clinical characteristic sub-groups

**Fig. 2:**
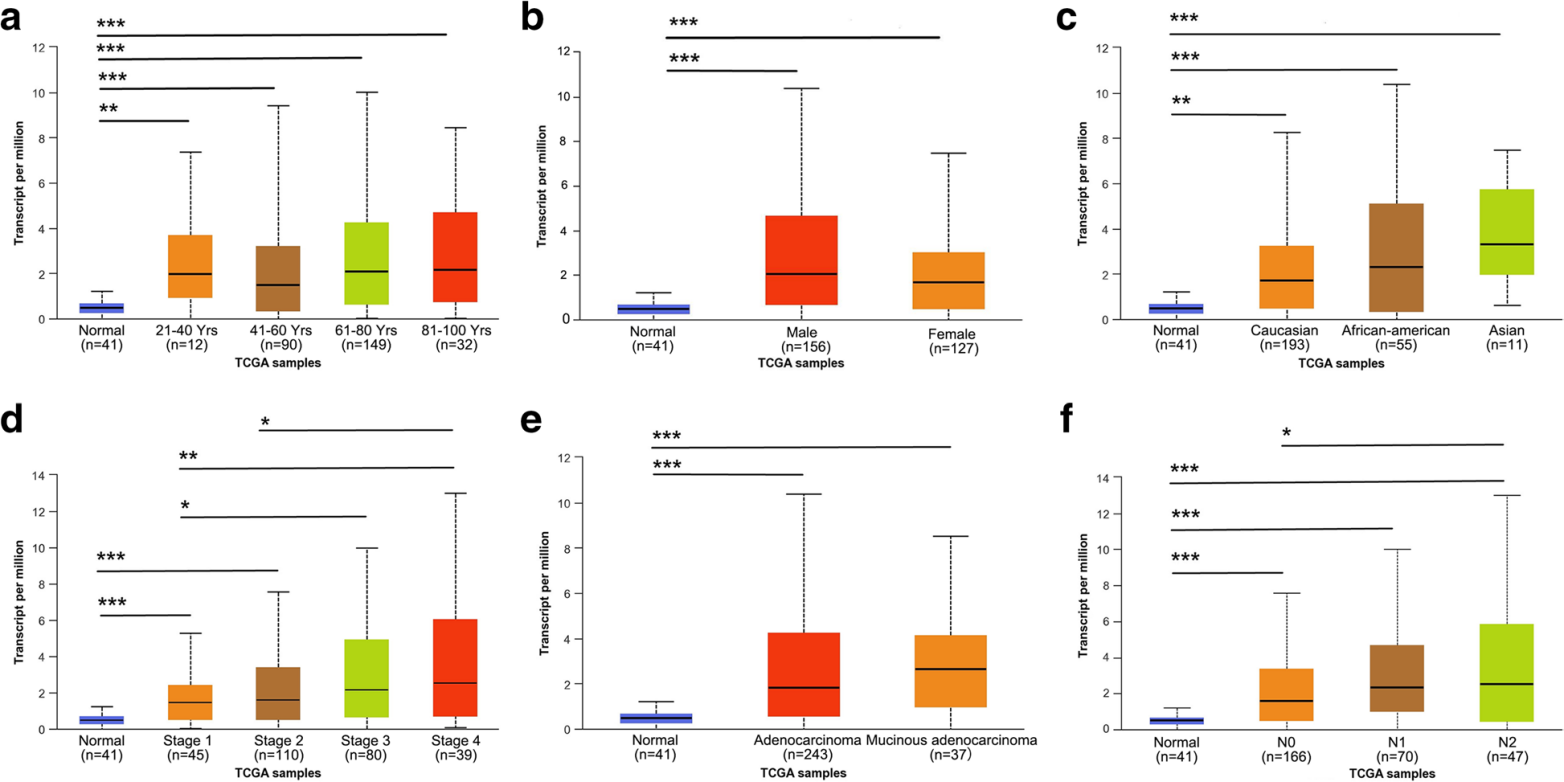
SRMS transcription levels in subgroups of patients with colon adenocarcinoma. **A** age, **B** gender, **C** race, **D** clinical stages, **E** histological types, **F** nodal metastasis status. Data are mean ± SE. *, *p* < 0.05; **, *p* < 0.01; ***, *p* < 0.001

**Fig. 3:**
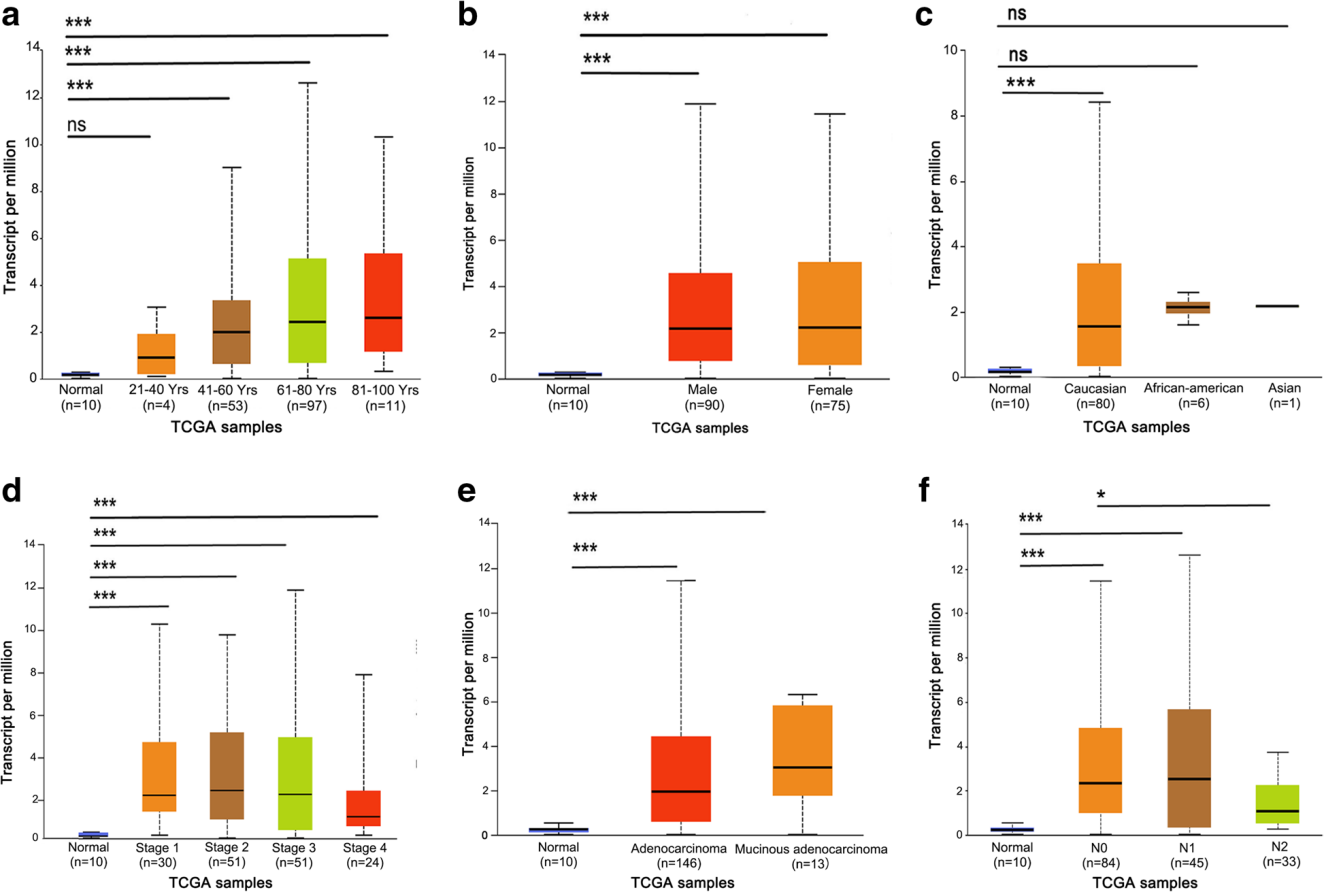
SRMS transcription levels in subgroups of patients with rectum adenocarcinoma. **A** age, **B** gender, **C** race, **D** clinical stages, **E** histological types, **F** nodal metastasis status. Data are mean ± SE. *, *p* < 0.05; **, *p* < 0.01; ***, *p* < 0.001; *ns* (non-significant), *p >* 0.05

The association between SRMS and clinicopathological features was further evaluated in the online cancer OMICS database of UALCAN. In subgroup analyses based on age, gender, race, clinical stage, and histological and nodal metastasis status, the transcription level of SRMS was significantly elevated in CRC patients than in healthy individuals (*p* < 0.05, Figs. 2 and 3). In COAD patients, SRMS expression levels were positively correlated with clinical stages and nodal metastasis. The expression of SRMS in stage 4 was higher than in stage 1 and 2 (*p* = 0.0028, 0.015, respectively). The expression of SRMS in N2 was high than in N0 (*p* < 0.05). In rectum adenocarcinoma (READ) patients, SRMS expression was negatively correlated with nodal metastasis*.* The expression of SRMS in N2 was low than in N0 (*p* < 0.05).

### SRMS co-expression networks in colorectal cancer

**Fig. 4:**
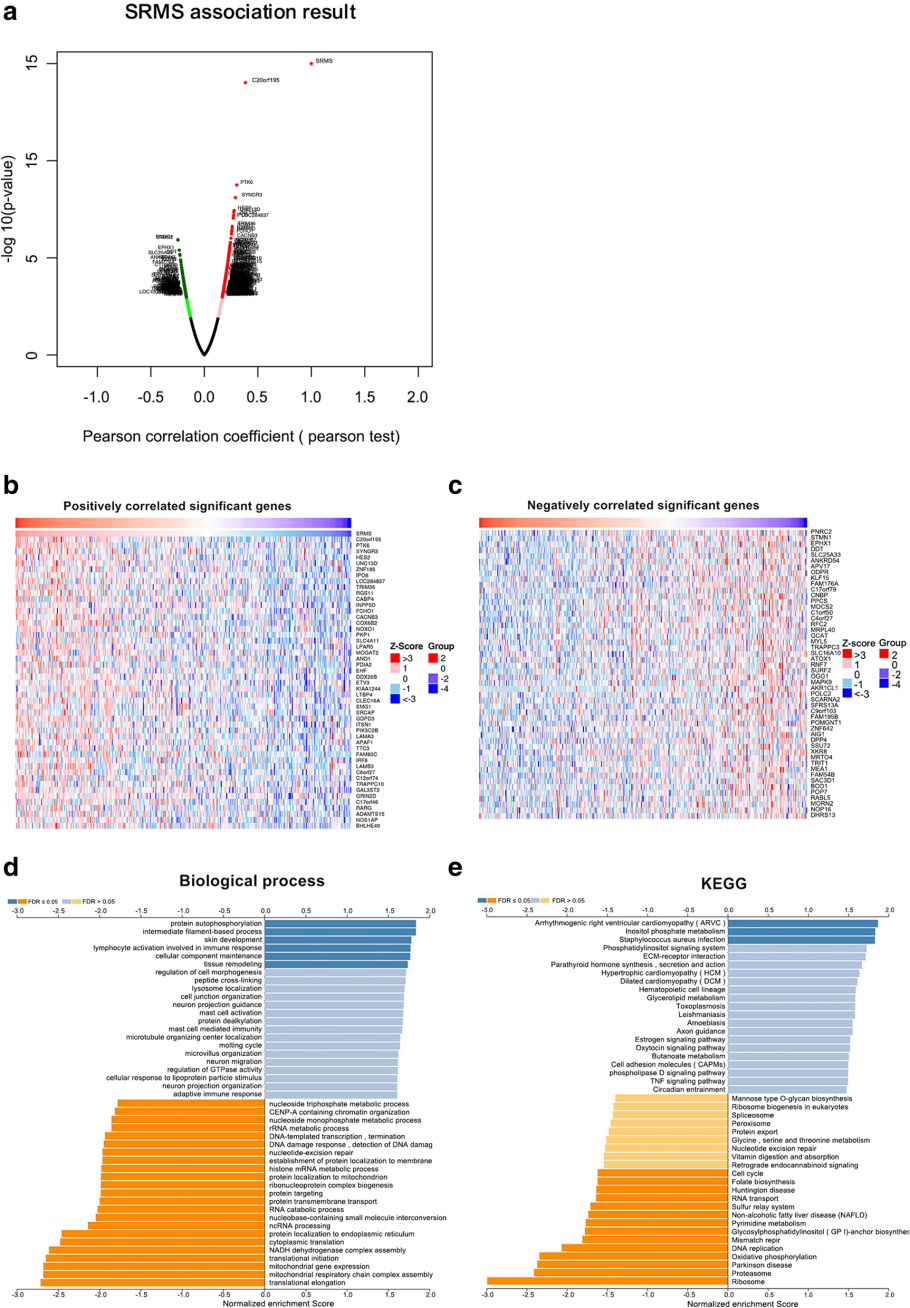
SRMS co-expression genes in CRC. **A** SRMS correlated genes in CRC were analyzed by the Pearson test. Red dots represent positively correlated genes dots while green represent negatively correlated genes. **B** Heatmaps of the top 50 genes that were positively correlated with SRMS. **C** Heatmaps of the top 50 genes that were negatively correlated with SRMS. **D** GO annotations: biological processes of SRMS co-expression genes. **E** KEGG pathways analysis of SRMS co-expression genes

To clarify on the functional properties of SRMS in CRC, the “LinkFinder” module in LinkedOmics was used to analyze the co-expression networks of SRMS. As shown in the volcano plot (Fig. 4A), a total of 2176 genes (red dots) were significantly positively correlated with SRMS while 1368 genes (green dots) were significantly negatively correlated (*p* < 0.05). The left and right heatmaps show the top 50 genes that were positively and negatively correlated with SRMS, respectively (Fig. 4B, C). SRMS expression was positively correlated with the expression of C20orf195 (positive rank #1, *r* = 0.389, *p* = 3.90E-15), HES2 (*r* = 0.301, *p* = 2.20E-9), PTK6 (*r* = 0.300, *p* = 3.04E-9), and SYNGR3 (*r* = 0.300, *p* = 2.43E− 9), and negatively correlated with the expression of PNRC2 (negative rank #1, *r* = − 0.247, *p* = 1.16E− 6), STMN1 (*r* = − 0.246, *p* = 1.20E− 6), and EPHX1 (*r* = − 0.234, *p* = 4.04E− 6).

GO biological process analysis by GSEA showed that co-expressed genes were mainly involved in the intermediate filament-based processes, epidermis development, and protein autophosphorylation (Fig. 4D). In contrast, translational initiation and elongation, mitochondrial gene expression, and cytoplasmic translation were inhibited. KEGG pathway analysis by GSEA showed enrichment in inositol phosphate metabolism, ribosomes, proteasomes, oxidative phosphorylation, and DNA replication among others (Fig. 4E).

### Association between SRMS and immune signatures

**Fig. 5:**
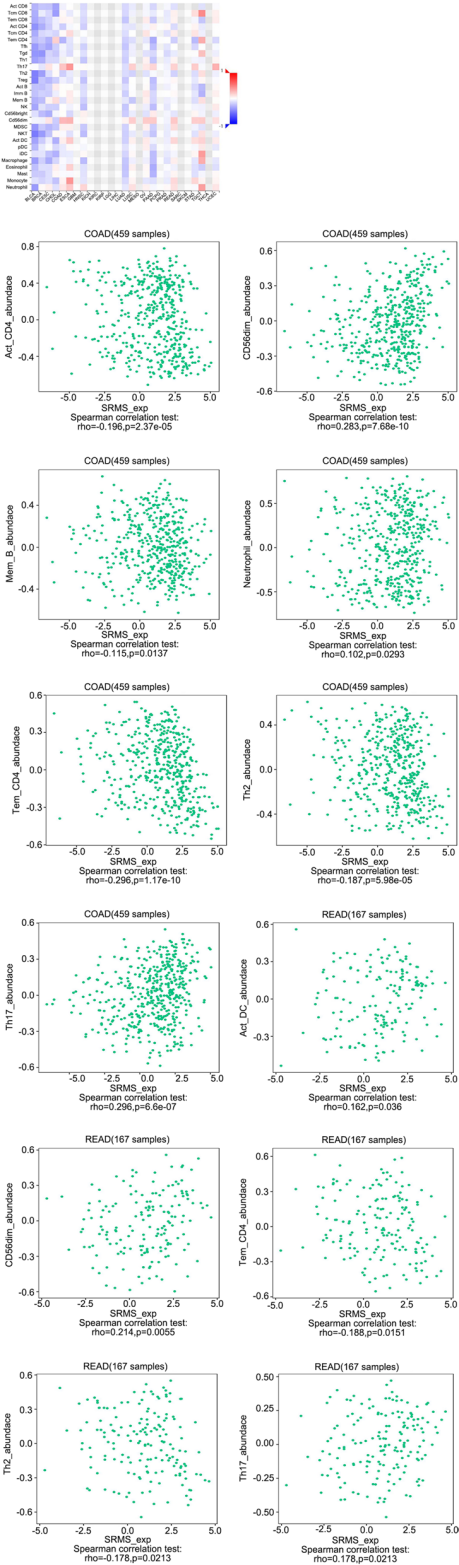
Correlations between SRMS expression levels and tumor-infiltrating lymphocytes. Dot plots show the correlations between SRMS expression levels and tumor-infiltrating lymphocytes in COAD and READ cohorts

Figure 5 shows that some immune subsets were associated with SRMS mRNA expression levels in COAD and READ. The 7 types of tumor-infiltrating lymphocytes that exhibited important correlations with SRMS in COAD included activated CD4+ T cells (Act CD4; Spearman: *r* = − 0.196, *p* = 2.37e− 05), CD56 dim natural killer cells (CD56 dim, Spearman: *r* = 0.283, *p* = 7.68e− 10), memory B cells (MEM B, Spearman: *r* = − 0.115, *p* = 0.0137), neutrophils (neutrophil, Spearman: *r* = 0.102, *p* = 0.0293), effector memory CD4+ T cells (Tem CD4, Spearman: *r* = − 0.296, *p* = 1.17e− 10), type 2 T helper cells (Th2, Spearman: *r* = − 0.187, *p* = 5.98e− 05), and type 17 T helper cell (Th17, Spearman: *r* = 0.23, *p* = 6.6e− 07).

The 5 types of tumor-infiltrating lymphocytes that exhibited important correlations with SRMS in READ included activated dendritic cells (Act DC, Spearman: *r* = 0.23, *p* = 6.6e− 07), CD56 dim natural killer cells (CD56 dim, Spearman: *r* = 0.214, *p* = 0.006), effector memory CD4 T cells (Tem CD4, Spearman: *r* = − 0.188, *p* = 0.011), type 2 T helper cells (Th2, Spearman: *r* = − 0.187, *p* = 0.0213), and type 17 T helper cells (Th17, Spearman: *r* = 0.178, *p* = 0.021).

**Fig. 6:**
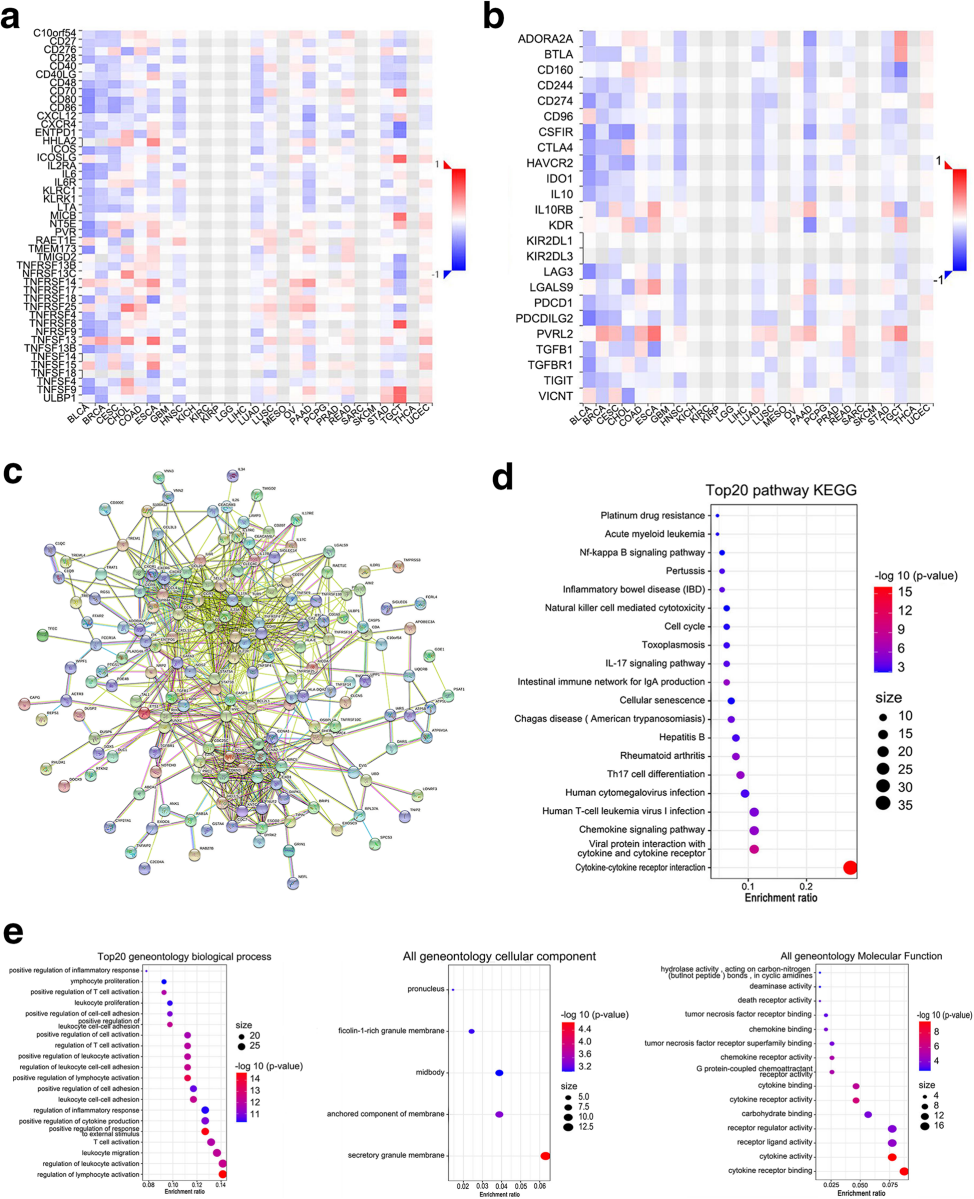
Identification and analysis of immune signatures associated with SRMS. **A** The heatmap shows the correlation between SRMS and immune-stimulators. **B** The heatmap shows the correlation between SRMS and immune-inhibitors. **C** Protein-protein interaction network of SRMS-associated immunomodulators and related immune cell marker genes in CRC. **D** and **E** are the KEGG and GO of SRMS-associated immunomodulators and immune cell marker genes, respectively, where larger dot sizes are related with higher gene counts and a darker red color is correlated to lower *P* value

Moreover, we identified 19 immunostimulators (C10orf54, CD276, CD28, CD70, CXCL12, ENTPD1, IL6R, KLRK1, RAET1E, TMRM173, TMIGD2, ULBP1, TNFRSF13B, TNFRSF14, TNFRSF18, TNFRSF25, TNFRSF4, TNFSF4, and TNFSF9) and 8 immuno-inhibitors (ADORA2A, BTLA, CD160, KDR, LGALS9, PVRL2, TGFB1, and TGFBR1) that were significantly associated with SRMS in CRC (Fig. 6A, B).

### Immune signatures associated with the SRMS gene

The SRMS-associated 27 immunomodulators and 191 immune cell marker genes were analyzed in the STRING database to validate functional connectivity. From the STRING database, we obtained the SRMS-associated immune gene PPI network (enrichment *p* value < 1.0e− 16) of a total of 214 nodes and 829 edges, which represented proteins and functional interactions (Fig. 6C). Next, we performed GO and KEGG enrichment analysis for these genes. The GO terms within the biological processes (BP), cell components (CC), and molecular function (MF) categories are shown in Fig. 6E. GO analysis showed that these genes were mainly enriched in the immune microenvironment molecule-related signals. Consequently, we probed the signaling pathways through which SRMS regulates immune responses in CRC. Figure 6D shows the top 20 enriched KEGG pathways, including cytokine-cytokine receptor interaction, chemokine, Th17 cell differentiation, IL-17, and intestinal immune network for IgA production. These signaling pathways may be related to SRMS-mediated immune events.

## Discussion

It is important to understand the basic mechanism of cancer occurrence at the gene level for increasing the efficiency of cancer therapy. In the present study, SRMS was highly expressed in CRC tissues compared to paired normal tissues.

A higher SRMS expression level was significantly associated with late TNM stages, more lymph node metastasis in COAD, but not with age, gender, race, histological. In READ, there was no significant association between the SRMS expression and patients’ age, gender, race, clinical stage, and histological. Moreover, SRMS expression was correlated with many immune cells and immunostimulators, and these immune signatures were focused on inflammation and cancer signaling pathways.

Tumor occurrence is closely associated with cell proliferation, differentiation, and apoptosis. Non-receptor tyrosine kinases, including PTK6 (protein tyrosine kinase 6), FAK (focal adhesion kinase), and Jak (janus kinase) are involved in cell differentiation, apoptosis, and proliferation through their interactions with transmembrane receptors [18]. PTK6 is the most studied member of the non-receptor tyrosine kinase family. Important roles of PTK6 in various cancers, including breast, prostate, and colon cancers have been reviewed [19]. SRMS and PTK6 genes are closely linked on the chromosome. Additionally, SRMS has been shown to biochemically interact with PTK6, which phosphorylates the C-terminus of PTK6 [20]. It has been reported that PTK6 expression is highest in normal colon epithelial tissues and decreases during colon tumor progression [21].

In the present study, we found that proteins, CNVs, and mRNA expression levels of SRMS were significantly elevated in CRC than in normal colorectal tissues. Copy number variants play a significant role in genetic variations and evolution, and can also cause genetic diseases and cancer [22]. We also found that elevated SRMS expression levels were correlated with poor prognostic outcomes, which may be involved in CRC progression. In addition, high expression levels of SRMS have been associated with advanced clinical stage and lymph node metastasis in COAD. However, SRMS expression was negatively correlated with nodal metastasis in READ*.* There are probably several reasons for this pattern: (1) The differences in anatomical location and biological function between COAD and READ. Studies have shown that right colon cancer patients had significantly higher gene mutation than left colon and rectum cancer patients [23]; (2) With lymph node metastasis, the exertion effects of some tumor suppressor factors reduce the expression of SRMS; (3) The number of cases with READ in UALCAN is relatively small. A larger sample size would obtain more robust and reliable statistic. Therefore, more clinical cohorts are needed to validate SRMS as a diagnostic or prognostic marker in CRC.

Within the gene co-expression network of SRMS, we identified genes that were significantly positively correlated, such as C20orf195, HES2, PTK6, and SYNGR3. C20orf195, also known as fibronectin type III domain-containing 11, is mainly expressed in the testis. Regrettably, its role in human disease is still unknown. HES genes are Notch downstream target genes, which could reflect expression levels of Notch signals [24]. Aberrant activation of Notch signaling has been associated with various tumors, such as breast and colorectal cancers [25, 26]. As mentioned earlier, PTK6 and SRMS have a close relationship. PTK6 is mainly expressed in epithelial tissues, with the highest levels in gastrointestinal linings [27]. In the azoxymethane model, PTK6 promoted STAT3 activation to promote survival and proliferation of damaged cells and colon tumorigenesis [28]. SYNGR3 is a synaptic vesicle-associated protein that interacts with the dopamine transporter. There are studies indicating that SYNGR3 may serve as a potential biomarker for the diagnosis and treatment of malignant tumors [29, 30].

We also found that SRMS expression was negatively associated with PNRC2, STMN1, and EPHX1 levels in the co-expression network. PNRC2, belonging to the PNRC family, was first found in breast cancer. In colorectal cancer, PNRC2 is expressed at low levels and PNRC2 upregulated can inhibit cell proliferation, migration, invasion, and EMT [31]. STMN1 is a major cytosolic phosphoprotein that regulates cell spindle formation and microtubule dynamics. Overexpression of STMN1 blocks the invasion of cancer cells and induces cancer cells growth arrest at the G2/M phase checkpoint [32, 33]. EPHX1, which mainly localizes in the endoplasmic reticulum, is a biotransformation enzyme. However, EPHX1 has been less studied in tumors. It is considered to be well associated with the anti-epileptic drug resistance [34].

In addition, co-expression functional networks were found to be mainly involved in protein autophosphorylation, translational initiation and elongation, mitochondrial gene expression, and cytoplasmic translation. Thus, the SRMS co-expression network is involved in post-transcriptional regulation, which is closely associated with protein translation and phosphorylation.

KEGG analysis showed the SRMS co-expression genes mainly enrichment in the ribosomal, proteasomal, oxidative phosphorylation, and DNA replication pathways. Ribosomes, comprised of ribonucleoprotein and non-coding ribosomal RNAs in eukaryotes, are conserved molecular structures required for protein synthesis [35]. Cancer development and progression are associated with ribosomal dysregulation, which affects the expression of key factors involved in tumorigenesis [36]. It has been reported that a single ribosomal assembly factor promotes lung adenocarcinoma progression through the Notch signaling pathway [37]. The proteasome pathway is one of the major mechanisms of protein degradation. Among these mechanisms, 26S proteasomes are the most active isoforms that are involved in cell cycle progression, apoptosis, and transcription [38]. Therefore, they are potential targets for anticancer therapy. Suppressed oxidative phosphorylation is a basic feature of tumor cells and tumors [39]. Huang et al. showed that LYRM2 directly interacts with complex I and enhances its activity, thereby promoting oxidative phosphorylation to induce colorectal cancer cell growth [40]. Since co-expressed genes share functions and affect each other, SRMS may play a role in CRC occurrence and progression through the above factors.

In the TISIDB database, we found that the Reactome pathway of SRMS was mainly involved in the immune system. Moreover, SRMS expression levels were positively correlated with infiltration levels of CD56dim, neutrophils, and Th17 and negatively correlated with Act CD4, MEM B, Tem CD4, and Th2 in CRC. There were studies that some TILs can promote *lymph node* invasion [41]. Moreover, tumor-infiltrating immune cell patterns are associated with cancer initiation and prognosis. For instance, neutrophils and Th17 have been shown to correlate with a poor prognosis in colorectal carcinoma [42, 43]. Given that *TILs* are critical for immunosurveillance and immunotherapy, SRMS potentially serves as a promising target to shape the immune microenvironment in colorectal cancer. In addition, we identified the immunomodulators that were significantly associated with SRMS in CRC. Tumor cells utilize various immune escape mechanisms, creating a microenvironment that is favorable for tumor growth and metastasis [44]. Immunomodulators have the potential for cancer treatment.

The biological functions of these SRMS-associated immune genes were explored using GSEA enrichment analyses. The enrichment of GO biological processes suggested that these genes mainly regulated immune microenvironment molecules by affecting their activation, migration, and adhesion, etc. Moreover, cell components showed that these genes were enriched in various membranes such as secretory granule membrane and anchored component of membrane. In addition, molecular function analysis showed that these genes chiefly regulate cytokines, receptor, and bind with various structures, such as carbohydrate, chemokine, and tumor necrosis factor receptor superfamily.

KEGG pathway analysis of SRMS-associated immunomodulators and immune cell marker genes revealed that cytokine-cytokine receptor interaction, chemokine, Th17 cell differentiation, IL-17, and intestinal immune network for IgA production may be involved in SRMS-mediated immune responses. Cytokine-cytokine receptor interaction is related to the viability and apoptosis rate of colon cancer cell lines [45]. It has been reported that the CXCL5 chemokine enhances the migratory and invasive properties of colorectal cancer cells by inducing epithelial-mesenchymal transition [46]. Chemokine signaling systems play critical roles in either the promotion or inhibition of tumor growth, proliferation, angiogenesis, or metastasis [47]. Aberrated activation of intestinal immune networks for IgA production signaling pathway promotes tumorigenesis [48]. IL-17 promotes tumor development through chronic tissue inflammation signals. In ETBF-colonized Min-CD4^Stat3−/−^ mice, IL17 blockade can significantly reduce colon cancer formation [49]. For many cancers, Th17-cell signatures (RORC, IL17, IL23, STAT3) are correlated with the worse clinical outcomes [50]. Therefore, it is biologically plausible that SRMS promotes tumor immunity by regulating multiple signaling pathways. This study provides the first evidence for the link between SRMS and tumor immunity, opening up more avenues for CRC research.

This study has several limitations. First, the data we analyzed were obtained from several public datasets, which is a lack of validation data. Second, the mechanisms of SRMS-mediates tumor immunity were not fully evaluated. More clarification and basic data are required to better assess the potential relationships between SRMS and CRC.

## Conclusion

In summary, the present study demonstrated for the first time that SRMS is overexpressed and associated with an adverse clinical outcome in CRC. Moreover, SRMS expression levels were significantly correlated with various immune signatures. These findings indicate that SRMS might play a role in the control of tumor immune microenvironments.

## Data Availability

All data supporting the conclusion of this article are included within the article.

## Abbreviations

SRMS: Src-related kinase lacking C-terminal regulatory tyrosine and N-terminal myristoylation sites
CRC: Colorectal cancer
GO: Gene ontology
TILs: Tumor-infiltrating lymphocytes
COAD: Colon adenocarcinoma
READ: Rectum adenocarcinoma
GSEA: Gene set enrichment analysis
KEGG: Kyoto Encyclopedia of Genes and Genomes
Act CD4: Activated CD4+ T cells
CD56 dim: CD56 dim natural killer cells
MEM B: Memory B cells
Tem CD4: Effector memory CD4+ T cells
Th2: Type 2 T helper cells
Th17: Type 17 T helper cell
Act DC: Activated dendritic cells
